# Hospital Medical and Nursing Managers’ Perspectives on Health-Related Work Design Interventions. A Qualitative Study

**DOI:** 10.3389/fpsyg.2020.00869

**Published:** 2020-05-05

**Authors:** Melanie Genrich, Britta Worringer, Peter Angerer, Andreas Müller

**Affiliations:** ^1^Institute of Psychology, Work and Organizational Psychology, University of Duisburg-Essen, Essen, Germany; ^2^Institute of Occupational, Social and Environmental Medicine, Centre of Health and Society, Medical Faculty, Düsseldorf University, Düsseldorf, Germany

**Keywords:** occupational health, work design interventions, evidence-based practice, healthcare, leadership, employee mental well-being, qualitative research

## Abstract

**Background:**

Research indicates that the active support of managers is essential for the sustainable implementation of health-related work design interventions in organizations. However, little is known about managers’ perceptions of such health promotion measures.

**Objective:**

Our study aims to provide information that help to foster managers active support of health-related work design interventions in hospitals. Based on Ajzen’s Theory of Planned Behavior (TPB) we explore the attitudes, perceived organizational norms, and perceived behavioral control of managers in the hospital regarding such interventions.

**Methods:**

Semi-structured interviews with 37 managers (chief physicians, senior physicians, and senior nurses) were carried out in one German hospital. A software aided qualitative content analysis was applied.

**Results:**

We observed that the majority of managers are aware of the importance of health-related work design. We found a high variation in the perception of organizational norms related to mental health promotion of employees. Behavioral control for supporting interventions is perceived more on an individual (e.g., appraisal interviews, professional development or support) and team level (e.g., fair work schedule, regular team meetings), less on an organizational level.

**Conclusion:**

To enable and to motivate hospital medical and nursing managers to support health-related work design, hospitals need to establish clear organizational norms that the health promotion of their employees is an important organizational goal. Moreover, managers need to get more work-design competencies and decision latitude to get more control. Important arguments for the top hospital management could be that health-related work design is highly effective for economic success, for treatment quality, and that the middle management already has a positive attitude toward the implementation of measures that help promote the mental health of their staff.

## Introduction

Physicians and nurses in hospitals are exposed to high work stress that puts them at risk for impaired well-being and health (Angerer et al., 2008; Pisljar et al., 2011; Burke et al., 2016; Coutinho et al., 2018). The Fourth European Working Conditions Survey reported, that in the European Union, 40 percent of employees in the healthcare sector suffer from constant health problems (Parent-Thirion et al., 2007). The recent Sixth European Working Conditions Survey shows that, compared to other professions, health care workers experience the highest work intensity, the most frequent interruptions, high emotional demands, and the highest exposure to social stressors, for example bullying, humiliating behavior or physical violence (Parent-Thirion et al., 2017). Beyond that, working conditions in hospitals are characterized by ongoing restructuring (Burke et al., 2016), demographic change (Halaweish and Alam, 2015) with aging employees (Weigl et al., 2011a, 2012), an increasing number of multimorbid patients (Warth et al., 2016), increased pressure on performance (Coutinho et al., 2018), and an increasing shortage of qualified workers (Goodin, 2003; Simoens et al., 2005). These developments show that working conditions in hospitals have become more stressful for employees than ever before.

Studies demonstrate that high workload, time pressure, work interruptions, high work demands with low control, mismatch between effort and return, insufficient social support or poor management style impair the mental health of employees in hospitals (Kivimäki et al., 2007; Angerer et al., 2008; Weigl et al., 2011b, 2014, 2016). Furthermore, impaired mental health of employees in hospitals can lead to intentions to lay off (West et al., 2009), an increased risk of presenteeism, sick leaves, decreased performance and even medical errors (Angerer and Weigl, 2015). Various studies provide evidence that burnout among health care professionals - caused by occupational stress - can endanger patient care: Empirical studies have shown that physicians with burnout are more likely to be involved in patient safety incidents (Shanafelt et al., 2010; Hall et al., 2016). High workload can lead to lack of professionalism that determines the quality of patient care (e.g., adherence to treatment guidelines, quality of communication). Unfavorable working conditions are also associated with lower ratings of patient satisfaction (Anagnostopoulos et al., 2012). In their review and meta-analysis, Panagioti et al. state: “Physician burnout is associated with suboptimal patient care and professional inefficiencies; health care organizations have a duty to jointly improve these core and complementary facets of their function” (Panagioti et al., 2018, p. 1318). In a meta-analysis, Zangaro and Soeken (2007) have examined the relationship between various variables on the job satisfaction of nurses. Occupational stress showed the highest correlation of all variables (Zangaro and Soeken, 2007).

Therefore, for hospitals the implementation of occupational health promotion interventions becomes increasingly important to ensure the well-being and employability of their staff, and to ensure the safe care of their patients. An essential part of occupational health promotion interventions, are health-related work design interventions, also called organizational or organizational-level interventions (Dahl-Jorgensen and Saksvik, 2005; Cox et al., 2010; Hasson et al., 2014; Montano et al., 2014). Health-related work design interventions aim to improve the working conditions of employees, based on established models of job stress, motivation and action regulation (Karasek, 1979; Hackman and Oldham, 1980; Semmer, 2006; Hacker and Sachse, 2014).

Notwithstanding the importance of such interventions, a significant lack of effective and well-evaluated health-related work design interventions in health care settings and beyond has been deplored for years (Richardson and Rothstein, 2008; Rugulies and Aust, 2019). Particularly, there is a need to better understand the design of intervention *processes* in order to effectively implement health-related work design interventions, and to develop complex systems like organizations (Saksvik et al., 2002; Murta et al., 2007; Nielsen et al., 2010, 2014).

Recent organizational studies point out the importance of the active support of managers for a successful implementation of such interventions (Nielsen, 2013). Using the Theory of Planned Behavior (TPB) (Ajzen, 1991) we assume that the active support of medical and nursing managers for health-related work design interventions in hospitals might strongly depend on their *attitudes*, perceived *organizational norms* and *behavioral control* toward such measures.

Against this background, the aim of our qualitative study is to examine medical and nursing managers’ perception of health-related work design interventions in the hospital based on the TPB. To the best of our knowledge, we present the first study that examines the perspective of hospital managers on such interventions. With this systematic qualitative analysis, our study aims to contribute to the further theoretical and conceptional underpinning of the design and successful implementation of much needed work design interventions in hospitals.

Available organizational research in this context is mainly focused on the *effectiveness* of health-related work design interventions, i.e., summative evaluation (Mikkelsen and Gundersen, 2003; Richardson and Rothstein, 2008; Montano et al., 2014). Moreover, success factors or obstacles to effectiveness as well as effective or faulty implementations are only considered *retrospectively*.

Recent studies suggest that the so far rather ambiguous results on the effectiveness of health-related work design measures can be explained by systemic or contextual factors (Nielsen and Randall, 2012; Nielsen et al., 2014). Therefore, currently, the focus of research is increasingly shifting from a mechanistically oriented input-output perspective to a systemically oriented context- and *process-oriented perspective* (Saksvik et al., 2002; Murta et al., 2007; Nielsen et al., 2010, 2014). The question is *how* to change workplaces and job characteristics to improve employees’ well-being. To analyze and understand these process factors adequately, appropriate theoretical models for evaluation and implementation research are needed (Kristensen, 2005; Nielsen and Randall, 2012; Nielsen and Abildgaard, 2013). In other words, research wants to know, what processes and structures are necessary to design “better jobs” (Nielsen and Abildgaard, 2013). Recent, systematic reviews take up this perspective to examine the context and process factors of health-related work design interventions, particularly the role of managers (Nielsen and Abildgaard, 2013; Müller, 2016; Daniels et al., 2017).

Current implementation research clearly shows that support from managers is one of the key factors for the success or failure of organizational interventions (Dahl-Jorgensen and Saksvik, 2005; Bourbonnais et al., 2006, 2011; Mattila and Elo, 2006; Nielsen et al., 2006; DeJoy et al., 2010; Petrou et al., 2016; Lundmark et al., 2017). Studies identify different forms of managerial support for organizational interventions: Managers have to support interventions actively and continuously. A lack of support from operational managers is mentioned as one of the main problems in the implementation research (Bourbonnais et al., 2006, 2011). Managers have to embed interventions in existing organizational structures. The structural secure of measures is needed before starting the implementation (Mattila and Elo, 2006; Nielsen et al., 2006; DeJoy et al., 2010). Managers have to establish a consensus on goals, opportunities, and limits of interventions to avoid critical side effects (Dahl-Jorgensen and Saksvik, 2005). Managers must provide time and human resources to enable execution of the interventions (Bond and Bunce, 2001; DeJoy et al., 2010). Managers should inform their employees about the intervention, communicate intervention goals in a motivational way, and allow their employees to participate (Petrou et al., 2016; Lundmark et al., 2017). Finally, managers have to decide whether to implement the developed work design measures.

These findings are important because organizational interventions that reported no, moderate or indifferent effects often argue with a lack of support from managers (Bourbonnais et al., 2011; Nielsen, 2013; Müller, 2016). In this study we assume that the TPB (Ajzen, 1991) will provide us with valuable insights and a more theoretically substantiated understanding of the role of managers in the implementation of organizational interventions.

### Theoretical Model: Theory of Planned Behavior (TPB)

The Theory of Planned Behavior (TPB) (Ajzen, 1991) as the extension of the Theory of Reasoned Action (Fishbein and Ajzen, 1975, 2015) is one of the most extensive studied models of human behavior and is used in a wide range of health and social-behavioral contexts (Godin and Kok, 1996; Armitage and Conner, 2001; Conner and Sparks, 2005). The TPB assumes that *attitudes*, *subjective norms and perceived behavioral control* (PBC) are predictors of behavioral *intention*. The intention acts as a mediator between attitudes, subjective norms, as well as PBC, and the dependent variable *behavior*. Besides, PBC is assumed to have a direct effect on behavior. Attitude is considered as the conviction of a person that a behavior leads to a consequence that is evaluated as positive or negative. Subjective norms are the “perceived social pressure to deal with behavior or not” (Fishbein and Ajzen, 2015). PBC is the perceived ease or difficulty and/or controllability to conduct a behavior, depending on internal and external factors. According to the TPB, a persons’ intention to behave is given when he or she has a positive attitude toward the goal of the behavior, perceives corresponding social norms, and perceives a behavioral control to carry out the behavior successfully (Ajzen, 2002). In this vein, we expect that a manager supports health-related work design measures, if she or he considers such measures as helpful, if the organization is perceived to place a great value on health promotion, and if she or he thinks that working conditions can be improved.

In a meta-analytical review of 185 independent studies, Armitage and Conner (2001) found that 27% of the variance in behavior and 39% of the variance in intention can be explained by attitudes, subjective norms and PBC (Armitage and Conner, 2001). In particular, it was found that PBC has significant explanatory power for intention and behavior, while the subjective norm has the lowest explanatory power. Overall, it was found that intentions can be predicted with high accuracy from attitudes, subjective norms and PBC. And intentions, together with PBC, have a predictive effect on the behavior itself (Ajzen, 1991).

Based on the above-mentioned findings we consider the TPB model as particularly well suited for our research context. It can represent the perceptions of managers concerning the implementation of health-related measures to support the mental health of employees. The TPB has not yet been taken up frequently in organizational contexts. There are first studies that make use of the theory to examine the intentions of employees to turn over or employees’ career choice and development behavior (van Breukelen et al., 2004; Arnold et al., 2006). Others use the theory in the context of organizational safety climate (Avci, 2014) or for planning health-related behavioral trainings (Bartholomew et al., 2006).

We identified three quantitative studies using TPB in the context of health-promoting interventions at work (Downey and Sharp, 2007; Wilde et al., 2011; Röttger et al., 2017). Two studies report the perspective of managers with the responsibility for implementing or promoting health-related measures in the workplace (Downey and Sharp, 2007; Wilde et al., 2011). Downey and Sharp (2007) stress that there are different groups of managers with different roles and different areas of responsibility. In their study the differentiate between general managers (GM) and human resource managers (HRM). GMs are responsible for formulating corporate strategies and decide whether employee health is part of them. HRMs are responsible for the planning and controlling of personnel and manage occupational health programs in companies. In order to sensitize managers for health-promoting measures, the authors recommend the development of programs that are appropriate for these specific management target groups.

Besides, Downey and Sharp (2007) extend the TPB model by another predictor, the “moral responsibility.” In this additional variable, they examined the managers’ personal moral obligation toward the well-being of employees. The study showed significant effects of moral responsibility on health-promoting behavior and interactions with “behavioral control.” While GMs are significantly motivated by moral responsibility, HRMs are not, but are significantly influenced by behavioral control (Downey and Sharp, 2007). By adding this further predictor, Downey and Sharp (2007) follow recommendations of Ajzen (1991), according to whom the TPB is fundamentally open to include additional predictors to increase significantly variance in behavior. Ajzen (1991) himself also acknowledges that moral components can influence intentions in the same way as attitudes, subjective (social) norms and PBC. We therefore have integrated this moral component as subcategories (see section “Belief in Role” and “Belief in the Importance of Mental Health”) of the category “attitude,” similar to the study of Wilde et al. (2011). Wilde et al. (2011) examined the conditions under which managers are more likely to show health-promoting leadership behavior. They refer to the 3 TPB predictors (*attitude, organizational norms, and PBC*) and differentiate the attitude component into an individual factor (“personal attitude”) and an organizational factor (“perceived outcomes on the employees’ health”). The “personal attitude” is described by items related to moral responsibility (e.g., “The health of my employees is very important to me,” “The health of my employees is more important to me than the achievement of given company goals,”and “I believe I am responsible for the health of my employees as a manager”). Based on the TPB, it is shown that it seems important to address individual as well as organizational factors if health-promoting leadership in companies should be strengthened.

The third study (Röttger et al., 2017) examines the participation behavior of employees in health-related interventions in organizations. The aim of this study was to derive recommendations for managers to increase the participation of employees. The results are broadly compatible with the assumptions of TPB that health-related behavior or respective intentions can be explained by the three variables attitude, subjective or organizational norms and PBC.

All three studies show that attitudes significantly predict the intention to support or participate in health-promoting measures. If managers or employees perceive a benefit, it is more likely that they will be committed to health-promoting work design measures. Concerning organizational norms, the studies report slightly different results. Wilde et al. (2011) assume that organizational norms (described as a “culture of healthy leadership”) are so strongly prescriptive for managers that they directly influence health-related leadership behavior. They report a high correlation between organizational norms and health-related leadership behavior and appeal to a detailed examination of this component in organizational studies. In contrast, Röttger et al. (2017) found a small negative effect of organizational norms on employee’s intentions to participate in health-promoting measures. The authors explain this finding with an effect of “psychological reactance” (Brehm, 1966; Brehm and Brehm, 1981) which in this context means that the perception of employees that existing organizational norms dispossess them from making free decisions can lead to defensive reactions. In addition, all three studies indicate that organizational (external) factors such as time, personnel or financial resources or a “lack of power to commit resources” (Downey and Sharp, 2007) moderate the direct influence of PBC on behavioral outcomes such as health-related behavior, the implementation of work design or participation in health-related interventions. This latter finding indicates in accordance with the TPB that external resources are required to effectively exercise PBC.

Based on these previous findings (Downey and Sharp, 2007; Wilde et al., 2011; Röttger et al., 2017), we assume that hospital managers are more likely to support organizational health promotion measures if:

(1) They consider organizational mental health promotion to be important and feel morally responsible for it (*Attitude*).
(2) There are *organizational norms* that to place a great value on health promotion. However, the role of *organizational norms* is not completely unambiguous, as they might also lead to reactance.
(3) They think that they possess internal or external resources to improve working conditions (*PBC*).

**FIGURE 1:**
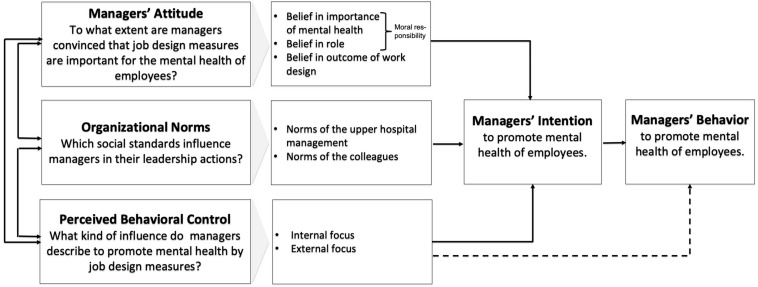
Research model based on the Theory of Planned Behavior (TPB; Ajzen, 1991).

Accordingly, we developed our research questions, based on the three predictors of the TPB *Attitude, Organizational Norms* and *PBC* shown in Figure 1.

The present interview study aims to fill a research gap by approaching the managers’ perspective toward mental health promotion by work design. While the previous studies on TPB in the context of health-promoting interventions at work focused on individual-related interventions or individual leadership behavior, we focus on the role of leaders in the implementation of organizational level work design interventions. Moreover, especially with regard to hospital managers, there seems to be a research gap, as we have not been able to identify a study that includes this group of managers. In line with existing studies, we hereby capture the specific perspectives of medical and nursing managers to take into account the differing roles and responsibilities of different groups of managers (Downey and Sharp, 2007).

Considering the so far sparse research on that topic we believe that a qualitative design will be particularly suitable for further theory building, as its openness and flexibility allow a better insight into the subjective perspective of the respondents and their patterns of thought and interpretation.

## Methodology

### Study Design

We conducted semi-standardized individual interviews in German language with upper medical managers (chief physicians), middle medical managers (senior physicians) and middle nursing managers (senior nurses) of a German hospital with two locations, belonging to a larger corporate organization. The larger clinic has approximately 500 beds and employs approximate 700 physicians and nursing staff. The smaller one has around 350 beds and about 450 medical and nursing employees and was converted from a specialized clinic to an emergency/casualty hospital.

In German hospitals a chief physician is the head of a department within a hospital (e.g., surgical department, psychiatric department). She or he is responsible for personnel management, the coordination of patient treatment, and budgeting in her or his department. Beside this management tasks a chief physician also participates directly in the patient treatment. The proportion of management tasks is about 70% in bigger hospitals, only 30% in smaller ones (Knüppel et al., 2006). The hospital of our study is one of the larger ones. The interviewed senior physicians officially represent the chief physicians. They have a “sandwich position” between chief physician and assistant or specialist physicians as well as hospital staff (Schmitt and Krasko, 2020). Senior physicians are directly subordinate to the chief physicians. They are the heads of smaller departments within the larger departments such as the surgical and psychiatric department. In comparison to the chief physicians, they take on more clinical tasks, as well as personnel management tasks. Senior nurses organize the processes within a nursing unit. Their main tasks include coordinating nursing care as well as economic and personnel management issues (Roloff, 2019). Physicians are not professional superiors of nursing but decide on medical treatment. For better readability, we refer in the following to the term manager when we refer to all three groups.

Our interview study has been proven by the ethic committee of the University Düsseldorf. In addition, we informed the hospital’s works council and the data protection authorities about the contents and the course of the study. All consents were available before the interviews were conducted.

We present the key results of the interviews along with the category system described in Table 1. Based on the TPB have deductively analyzed the three main categories: Managers’ *Attitudes*, *Organizational Norms* and *Perceived Behavioral Control (PBC).* Inductively we have divided each category into further subcategories.

**TABLE 1:** Category system for interview analysis.

**Managers’ Attitude**	**Definition research question**	**Anchor sentence examples**	**Inclusion criteria: Related issues**	**Exclusion criteria**
Belief in importance of mental health	The managers’ belief in the importance of employee mental health.	The mental health of employees is a very important issue for me, which is neglected in everyday life. But there is a great need for it (SP 37).	How important is the mental health of employees in your hospital? −>Own attitude to the topic	Attitudes that extend beyond health-promoting leadership behavior. Indirect formulations that are subject to interpretation.
Belief in role	The manager’s belief in whether the promotion of employee health is the responsibility of the manager.	It is not only the task of the supervisor to pay attention to the employee’s health, but also vice versa. We do not differentiate, we are all involved in people, whether nurses in training or senior physicians (SP 37).	How would you describe your role as a supervisor for the mental health of your employees at work?	
Belief in outcome of work design	The manager’s belief that work design measures have a positive or negative effect on the health of employees.	For me, it is a quality if we can openly communicate mistakes, uncertainties or the need for support. I believe that working in flat hierarchies also makes us better as a team (SP 34).	What stressors are the most important and which working conditions do you consider supportive and motivating for your employees? Do you see a connection between the stressors you have just mentioned and the mental health of your employees?	
**Organizational Norms**				
Norms of the upper hospital management	Perceived social pressure or organizational standards of hospital management that influence managers in their behavior to promote mental health.	I feel that the issue of mental employee health is not a priority for the upper hospital management (SP 33)	How important is the mental health of employees in your hospital?	Statements with managers’ own attitude to the topic.
Norms of the colleagues	Perceived social pressure or organizational standards of colleagues that influence managers in their behavior to promote mental health.	I think it’s very important to everybody. I think that people deal with it in very different ways (CP 07)	What opinions do your colleagues have on the subject?	
**Perceived Behavioral Control**				
Internal focus	The manager’s experience of self-efficacy and/or sense of control for the implementation of work design measures by his own resources.	It motivates employees when you give them confidence and let them make their own decisions, but stand behind them (SP 38).	What changes do you think can be implemented to reduce the strain on your employees in their day-to-day work? What opportunities do you see for yourself to maintain the ‘mental’ health of your employees and to reduce the stressors you mentioned?	Statements related to employee activities.
External focus	The manager’s experience of self-efficacy and/or sense of control for the implementation of work design measures in connection with organizational possibilities and limits.	In the age of a shortage of skilled workers we are whistling from the last hole. I wish I could, but there’s no time for team reflection. What we can do to minimize stress. We don’t do this enough; we should do it more often (SP 37).		Statements related to employee activities. External factors that can be influenced by managers −>Internal factors

Concerning the variable *Attitude*, we examined if managers are convinced of the importance of work design measures for the mental health of employees. Additionally, we evaluated two further inductive ‘moral components’: the managers’ belief in the importance of mental health and their belief that responsibility for employee’s mental health is part of their leadership role. To understand which organizational standards influence managers’ leadership actions, we examined their perceived norms of the upper management and their colleagues.

As described above, we further assessed internal and external factors related with *PBC* to promote work design measures. In our study, *internal focus* includes on the one hand managers’ perception that they personally have the abilities, experiences or skills to implement health-related work-design measures (self-efficacy component). On the other hand, internal factors include managers’ perception that they can control the organizational conditions that are relevant for the implementation of measures (controllability component). The *external focus* means the managers’ perception of how supportive the organizational conditions are for the implementation of health-related work-design measures, or if they even hamper the implementation of such measures.

### Recruitment

The data collection took place from April to July 2018. We recruited chief physicians (upper management), senior physicians (middle management) and senior nursing staff (middle management).

Participation was voluntary but recommended by the hospital managing director, medical directors and nursing service management. The participants were allowed to conduct the interviews during their working hours. We recruited the participants in various ways: (1) Information about the interview study at meetings of chief physicians and senior nurses, (2) Sending participant information about the interview study and reminder by e-mail and, (3) Appointment coordination by telephone after expression of interest by the managers, partly via secretarial offices. We informed the participants about the data protection and privacy; participants had to sign a letter of informed consent before start of the interview. The interviews were carried out “Face-to-Face” within the hospital. It was almost always possible to conduct the interviews without interruption (e.g., through emergency treatment). By approval of the managers, the conversations were audiotaped. The interviews lasted on average 45 min. They were conducted by a certified pedagogue with systemic qualification, a psychologist and a medical student which is also an examined nurse. All interviewees explained their background and stated that there might be clarifying questions about specific professional issues. The expert role was assigned to the participants.

### Design of the Interview

We used an interview guide as a basis for the interviews. For introduction, we asked the managers about their perception of the most important organizational stressors and resources for their employees: *“What do you think are the most important work stressors for your employees?” “Which working conditions do you consider supportive and motivating for your employees?”* Based on the TPB (Ajzen, 1991), we asked the following questions to the variable “*Attitude”* toward health-related work design measures: “*Do you see a connection between the stressors you have just mentioned and the mental health of your employees? How would you describe your role as a supervisor for the mental health of your employees at work?*” In respect to the *“Organizational Norm*” according to Ajzen’s subjective norm, we wanted to know: “*How important is the mental health of employees in your hospital? What opinions do your colleagues have on the subject”* To assess aspects of “*PBC*” we asked the following questions: “*What changes do you think can be implemented to reduce the strain on your employees in their day-to-day work? What opportunities do you see for yourself to maintain the* “*mental*” *health of your employees and to reduce the stressors you mentioned?*”

A draft of the interview guide was discussed by the study team. It was afterward tested in an expert interview with a doctor from one university hospital and then slightly modified. The interview study was conducted by three interviewers of the study team. After the conduction of the first six interviews, we consolidated whether it was necessary to make further modifications. No changes to the guide were necessary. The interviews were conducted until the point of “theoretical saturation” was achieved. Glaser and Strauss defined this as points of analysis at which ‘no additional data are being found whereby the researcher can develop properties of the category. As he sees similar instances over and over again, the researcher becomes empirically confident that a category is saturated. When one category is saturated, nothing remains but to go on to new groups for data on other categories, and attempt to saturate these categories also’ (Glaser and Strauss, 1967, p. 61; Guest et al., 2016). After each interview, the interviewer reflected and documented the conversation and particular perceptions (atmosphere, disruptions, and communication problems) in the course of the interview. During the data evaluation, these recordings were compared to the evaluation results and taken into account in the interpretation.

### Data Analysis

The transcription of the digitally recorded interviews was acquired by a transcription office and then analyzed by the study team using structuring content analysis (Hsieh and Shannon, 2005; Elo and Kyngas, 2008; Mayring, 2015). This type of analysis pursues the goal to deductively summarize and systematize the contents of the interviews on the basis of theoretically derived dimensions in such a way that the results can be understood intersubjectively (Potter and Levine-Donnerstein, 1999; Creswell, 2014). Definitions, anchor examples, and coding rules were defined for all upper and subcategories (see Table 1). We tested the reliability of our coding rules in two steps: At first, as a formative reliability test, three members of the study team applied the coding guidelines based on three randomly selected transcriptions and then checked and discussed the results regarding similarities and differences. The results were discussed with the large study team including the project leaders. Slight modifications were made to the coding guide. This procedure was followed by a summative reliability test. For this purpose, 2 × 4 transcribed interviews were coded by one researcher and independently counter-coded by another researcher. Based on the results, the interrater reliability was calculated. A Cohens-Kappa value of 0.72 indicated moderate to good reliability of our coding system (McHugh, 2012). In the first review of the transcriptions, the statements of the managers were assigned to the described categories. In the next step, we analyzed the statements within each category for a possible more in-depth systematization. In this way, further subcategories were formed based (inductive approach). All subcategories are shown in Table 1. Meaning units were words, statements, and paragraphs which were assigned to the categories. The meaning units were condensed for reporting the interview results. The digital software MAXQDA 2018.1 was applied for the analyses.

## Study Results

### Sample

We interviewed 37 managers, including 23 medical professionals (14 chief physicians, CP, out of total 29; 9 senior physicians, SP, out of total 20) and 14 senior nurses (out of total 44). The interviewees work in different medical departments, shown in Table 2. The age of the participants ranged from 34 to 60 years.

**TABLE 2:** Sample of the study.

	**Chief physicians**	**Senior physicians**	**Senior nurses**
Number	14	9	14
Female	2	2	9
Male	12	7	5
Age range	43–60 years	38–60 years	34–60 years
Departe-ments	Anesthesia, dermatology, gynecology, vascular surgery, cardiology/intensive care medicine, pediatrics and juvenile medicine, hospital hygiene, hand and plastic surgery, pneumology and sleep medicine, radiology, spinal surgery, vascular surgery, psychiatry, urology, internal medicine.	Anesthesia, cardiology, neurology, pneumology and sleep medicine, spinal surgery, urology, hand and plastic surgery.	Oncology and hematology, pediatric and youth intensive medicine, anesthesia, occupancy management, sleep laboratory, internal intensive medicine, trauma surgery, general surgery, pediatrics and youth medicine, spinal surgery, geriatrics and psychiatry.

### Interview Results

#### Managers’ Attitude

##### Belief in the importance of mental health

In general, we found that the managers are sensitized to the importance of the mental health of employees. They place great importance on the topic of mental health in the hospital. Despite the high relevance, managers repeatedly stated that the topic is often neglected in their work routines. We did not observe major differences in the responses between the occupational groups.

*“I think the subject [mental health] is very, very important. Employees are reaching their limits*” *(CP 6). “From my point of view, the subject is extremely important. We bring in a lot of commitment to our job. In my understanding of practicing the job as a doctor, you have to feel just as comfortable in your job as in other areas of life*” *(CP 7).*

*“For me, employees’ mental health is a very important topic. Although it is neglected in everyday working life, there is a great need for it*” *(SP 37).*

“It’s important that we do a lot of mental hygiene in our team” (SN 16). “Altogether I find the topic interesting. It is never really discussed and is being missed out” (SN 17). “The issue [mental health] should be more openly discussed. Needing support should not have a negative connotation” (SN 24).

##### Belief in role

Almost without exception chief physicians, senior physicians and senior nurses feel responsible or co-responsible for the mental health of their staff, even if the top priority is good patient care. They see themselves in the duty of care, want to make sure that the employees are doing well or want to be a role model, which is being perceived as a troubling role conflict by some of them. Participants report, that it is not always easy to reconcile the demands of economic efficiency and the assurance of good patient care while at the same time being a good role model for employees. The personal work demands and responsibilities are often high, which is why everyone has to take care of everyone: managers for employees and vice versa.

“I also want the employees to feel good, I have a responsibility for them. I don’t want to exhaust the employees, but I also have to take the economic factor into consideration at the same time” (SN 26). “As the chief physician, I have to take on a role model function and at the same time I have to give clear instructions. I have never been ill in the past 15 years, but I cannot demand that from my staff” (CP 06). “It is my responsibility to keep an eye on the mental health of my employees. I am also responsible for ensuring that the finances are correct, but for me, medical care comes before economic interest” (SP 31). “It is not only the task of the managers to look at the employee, but also vice versa. We do not differentiate, we all work with people, no matter whether they are nurses in training or senior physicians” (SP 37).

Even if everyone feels responsible, managers describe differentiated understandings of their roles. These are dependent on the work situation and are reflected in examples of behavior. These roles range from more protective roles:

“*I believe that I have a very important role to play about this issue [mental health of employees]. I am more of a mom; I arrange or solve things. Sometimes I feel overburdened myself, but it’s my job to look after myself” (SN 29).*

or supportive roles:

*“12-h days have been the routine for me, since everyone knows that me, the boss is still on duty and it is possible to talk to me. If I promise employees something, then I want to be 100% sure that I can keep the promise”* (CP 07).

up to more promotive roles:

“I have to recognize where the boundaries of colleagues are, and expanding them. Demanding and encouraging are important aspects” (SP 36)

or demanding roles:

*“Medical care has to be at the top, it has to be guaranteed and sometimes you have to be very hard [to the employees]. I think I am a little bit more human” (SP 33)*.

Only one chief physician thinks that the responsibility belongs to each individual. He perceives himself rather helpless in the role of a manager. Another senior physician describes a common responsibility with a focus on occupational medicine and upper management.

“Mental health is the responsibility of each individual. I have little influence on it” (SP 08). “Every employee in a managerial position has a responsibility for the mental health of their employees. Everybody has to take care of the health of someone else, if someone notices a need and it is possible for him to act. The task lies in particular with the occupational physicians and in the broader sense with the upper management” (SP 32).

##### Belief in the outcome of work design

In general, we can report that managers are aware of the interdependencies between working conditions and the mental health of employees.

“You have to make the workplace attractive so that people also want to work here. If I am satisfied with my work or I have a structured schedule, then I am personally more relaxed. I am more balanced, stress-resistant, even if it is a lot of work” (SP 35). “Stress at work can make you sick. I think we have colleagues in our hospital where burnout is inevitable” (SP 38). Stress is caused by a lack of personnel and a 10% are due to poor organization’ (SN 18).

Managers mention a range of job characteristics or approaches of work design that they believe have an impact on the mental health of employees. They particularly describe interactional or social supportive approaches for health promotion in hospitals, like the assisting with tasks or employee appraisals. Less often approaches for structural work design are mentioned, like changing work tasks or work processes. Additionally, to the mental health-promoting effects, managers also mention motivational, economic or patient-related effects which they attribute to work design measures as well.

The following Table 3 shows the most frequently mentioned approaches to work design that are assumed to have a positive effect on health-related but also on motivational, economic or patient-related factors. Selected approaches are illustrated in the following by citation.

**TABLE 3:** Managers’ focus on health-related work design measures.

• Respectful and appreciative teamwork • Development of a functional team with flat hierarchical and social supportive structures • Appropriate distribution of tasks and job autonomy • Simplification and relocation of administrative tasks • Opportunities for occupational and personal learning and development • Functioning interdisciplinary cooperation, communication and workflows • Flexible working time models, staff-oriented shift schedules and break times • Meaningful work • Team justice • Good leadership behavior

The majority of managers describe health-related benefits of effective teams with social support structures. This social support can be individual or team-related.

“I think there’s a connection between work stress and mental health, that’s how it is. For instance, if the stress is perceived as too high or certain experiences cannot to be coped with well. If this is not discussed accordingly, of course, it can also affect health. Depending on how sensitive someone is” (CP 09).

“I take the number of absences due to illness as a value by which I recognize how satisfied and able to work employees are. A bad team atmosphere leads to a downward spiral. A central point for me is a functioning communication. It doesn’t work without it” (CP 05).

“A healthy climate promotes teamwork. When you realize that a team works, everything is much easier. If there is less pressure, then you have more ideas. The motivation is completely different” (SN 28).

In comparison to the other occupational groups, senior physicians more often mention the benefits of flat hierarchical structures. Some of them report from experiences of their own departments, others with a view to other departments of whom they believe that strong hierarchies are still existing.

“I believe in flat hierarchies. Strong hierarchies lead to dissatisfaction and stress. An important factor in a working relationship is that you communicate openly and fairly, regardless of hierarchies” (SP 31).

“It also has to do with mental health when I am dissatisfied because I am not seen or because my issues are not seen. That leads to dissatisfaction. It is shown by fluctuations in the departments. The flat hierarchies are doing quite good for employees’ satisfaction” (CP 02).

A functioning interdisciplinary cooperation, transparent communication and workflows are starting points for many managers to avoid stress for employees.

*“Due to the lack of communication, many problems are generated at the back. The time and effort are much higher because you do not clarify things directly. We would have to give ourselves this hour for exchange of information, e.g.*, *conduct consultations at the patient’s bedside in order to solve problems directly. If we carry the problems around with us, it is a burden” (SP 36).*

Another important approach across all occupational groups is the design of flexible working time models, staff-oriented shift schedules and break times. Even if there are managers who disagree, the majority of the interviewees agree that it is becoming increasingly important to develop working time models that are more focused on the work-life balance and lifespan of employees.

“I believe that attendance and shift work is not healthy for older employees” (SP 34). “Employees must also have periods where they can sleep thoroughly” (SP 33).

“If I constantly have to work in exhaustion mode, it affects my resilience. We’ve reached a limit that is often exceeded. Especially assistants do not have a lot of compensation range. That might endanger their health at some point” (CP 11).

“Today, you can recruit employees by offering financial incentives. However, it is even more important to consider the work-life balance of the employees. But developing employee and family oriented working time models requires money” (CP 06).

In general, the interviewees see a strong association between mental health or well-being with job satisfaction, job motivation, and productivity. Especially chief physicians describe the connection between mental health, employee’s motivation and increased productivity in the economic context. In addition to the human perspective, chief physicians take on a stronger functional perspective on the impacts of work design measures. Moreover, they believe that a healthy working atmosphere has positive effects on the attractiveness of the hospital as an employer, on reduced fluctuation of employees or an increase in work performance.

*“Fluctuations in the departments can be attributed to dissatisfaction of employees.* […] “*Satisfied employees are more efficient, less ill. The productivity of the hospital is increased. It is important to promote employees” commitment and trust. We have to show that something is done for them. Good training is one of the most important things. This is the only way I can attract employees, and we have to do so” (CP 02).*

“Mental health affects work input and the ability to work” (CP 05). “If I can generate a certain level of job satisfaction, employees work more optimistically and better” (CP 06).

“Stress affects the quality and quantity of work” (SN 26).

Some interviewees additionally mention patient-related effects of work design, like the better quality of patient care or the reduction of complaints.

“If we’re fine, the patients are better too” (SN 16). “Stress results from doing things in a very timely manner and the patient does not get enough care” (SN 20). “Mental health effects good patient care, the staff would be more motivated” (SN 24).

In a comparison of occupational groups, chief physicians more often establish functional connections between mental health and economic outcomes, which can be explained by the fact that they are in charge of budget responsibility and therefore are more strongly affected by financial-related role conflicts.

#### Organizational Norms

In summary, we found, that managers have very different perceptions of the *organizational norms* regarding mental health promotion in the hospital. Their views seem to depend on their previous experiences with how health related issues were handled by the management. The question about the importance of the topic mental health for the hospital is answered by the managers mainly by referring to upper hospital management (i.e., board of management), or to the nursing service management. Senior physicians seem to have a more negative view of *organizational norms* than the others.

##### Level of upper hospital management

Most physicians and nurses believe that the topic of mental health promotion does not have a high importance/value for the upper hospital management (board of management and nursing service management). Instead, they believe that financial priorities are at the center of attention of the upper hospital management. Some managers suspect, that the hands of upper hospital management are also tied when trying to improve the working conditions for employees. The pressure of the employees may be perceived by the upper hospital management, but there seems to be a lack of practical solutions or ideas.

“I don’t know if they [the upper hospital management] don’t want to or can’t see it because they might have instructions from the top [the corporate management] to save on personnel” (SN 21).

“No one at the upper hospital management seems to be worried about the subject [mental health]. Here one works with Excel-Sheets and it is important that the numbers are correct. I don’t think mental health has a high priority for them” (CP 15).

“In general, we think that we are left alone with the problem here. Nobody cares how the situation is going to develop. The stress level is known to the upper hospital management, the nursing service management, and the works council. But there are no real offers or measures of improvement” (SN 22).

Those managers who have been working in the company for some time emphasize that the financial pressure has increased with the takeover of the corporate organization. Others merely assume that mental health promotion must be a upper hospital management subject matter because it is such a pressing issue. But it is not open communicated.

“The subject [employees’ mental health promotion] is not openly discussed. The topic is rather a marginal topic for the hospital management, who probably don’t want to sting into a hornet’s nest. But I assume that the topic is also important to them if they want to change the number of sick leaves” (SN 26).

Some offers for employees’ health promotion are perceived by the interviewed managers, but they doubt whether they actually reach the employees. Some staff members introduce measures on their own initiative like running groups, etc.

“At the upper hospital management level there are certainly verbal efforts, but at the practical level, this is not seen” (CP 06).

It is assumed that health promotion measures are exclusively a matter of maintaining the work ability and performance. Some interviewees do not see any efforts from the upper hospital management at all. They think that the topic is ignored, and nothing is done. These managers describe a certain helplessness and frustration. More senior physicians than chief physicians seem to take this negative perspective. Some have the opinion that everyone has to deal with stress for themselves, it seems to be part of the job.

“No member of the upper hospital management seems to be worried about the topic” (CP 15).

“The topic is not actively addressed in the upper hospital management, it is suppressed. Perhaps for fear that it might be a sign of weakness” (SP 37). “I would say that the topic of mental health has no value for the hospital. Nothing is done. If there is an initiative, it is private one. Such as a running team. But the employer does not promote that” (SP 33).

“The subject is not really discussed. It also seems that you have chosen the wrong job if you cannot cope with the strain” (SN 17).

Few managers report that they have seen the upper hospital management as very supportive on the topic. They attribute this to their own personal experiences and report on situations in which they have experienced the upper hospital management as helpful.

“The topic of mental health is an important concern for the upper management in this organization. I have never experienced this before in other hospitals” (CP 04).

“I believe that the subject is becoming increasingly important. I have been here for a long time and have noticed that the hospital management and the nursing service management are interested in keeping the employees healthy” (SN 25). “The topic is considered. The main thing is that we are ready to work. We have a good relationship with our nursing service management” (SN 29).

##### Level of the colleagues

At this level, we observed a difference between the physicians and nurses. While the nurses are convinced that their colleagues also consider the topic important and are interested in it (although it does not seem to be formally discussed), some physicians report on contrary attitudes of their colleagues. Especially for that occupational group, not all of them seem to think that mental health promotion is important.

“I don’t think the topic of employees’ mental health is always on the agenda of every chief physician. There are always assistants who are unhappy because the boss does not take care of the department’s needs. To some extent, there are still the old, hierarchically shaped bosses. This has nothing to do with humanity” (CP 02). “In some departments, it will be a topic. There will be others that will repress the subject and some departments that are intact and well equipped with personnel so that it is not so bad” (CP 13).

“We’ve never thought about the topic before, but I think it is an issue. Just when the study was presented to us. in any case. The colleagues are thinking the same” (SN 16). “We are all in the same boat here. It is not about who is worse, who is better, but that we come forward together. That is already a very great cohesion here. Loyalty is also a big issue for us” (SN 27).

What unites the occupational groups is the fact that the issue is only discussed informally among the colleagues.

“*You can informally discuss it [mental health] with close colleagues” (SN 28). “There is an exchange, but more on a collegial, informal level” (CP 08).*

Therefore, it seems to be a sensitive issue that is given importance, but generally it is not communicated in an open, well-structured and solution-oriented way.

*“The matter [mental health promotion] is repressed, pushed away. When you are in a bad mood, and say “it totally sucks here,” there are no consequences. Nobody asks what they are supposed to change or how we can improve” (SP 35).* “*Internally, it is already talked about, but without structured thoughts. Our team is characterized by very conservative attitudes. You can’t discuss certain things constructively with some of the colleagues” (SP 38).*

#### Perceived Behavioral Control

The *PBC* is influenced by *internal* and *external* factors that moderate the indirect or direct influence of PBC on behavior. Therefore, we present the results from both perspectives. The interview results show that managers perceive mostly behavioral control to provide socio-emotional support at the individual or the team level. They report less behavioral control to change the work processes and work organization. While many managers report that they reach their limits due to the complex and restrictive organizational conditions, few describe organizational possibilities or ideas for better work design measures.

##### Internal focus on managers’ behavioral control

Managers are most likely to experience internal behavioral control in social supportive measures on individual contact or at the team level. They report that it is helpful for themselves if they are in good and direct contact with the employees and notice problems at an early stage: e.g., stressful treatment cases or team conflicts. They experience self-efficacy when they are aware of their employees’ problems, and can actively address them. To some extent it is the offer of professional social support, but it is also the social-emotional social support in which the managers experience themselves effectively.

“Sometimes it is already sufficient for employees to notice that we are aware of the existing demands. I have a good feeling for the satisfaction of my employees because we see each other in meetings every morning” (CP 13).

“I can’t assess the mental health of my employees. I’m not a psychologist or a counselor. It’s not my job either, I only have little knowledge of human nature. I can offer conversation for my employees. I don’t know everything, but if the employees have a problem, they can talk to me and I try to mediate. I can listen and give practical tips from an aging man. That’s all I can do. I think I’m doing quite well overall; I get perceived like this” (CP 15).

Some managers feel that it is a challenge to find a balance between supporting, demanding and encouraging their employees. Some consider it easier to relieve overworked employees, instead of helping them to cope with the demands for themselves. As a result, the managers must be careful not to reach their own limits.

“I can relieve the workload of my employees by taking the work off their hands. Sometimes I do so too quickly. That’s a fine line because you want to teach something and not be used for it. I have to delimit myself as a senior physician. Caring on one hand and not being exploited on the other hand. That’s difficult” (SP 38).

*“We are raised with a helper’s syndrome, which isn’t always good. But I can’t save everyone”* (SN 18).

Some interviewees report, that they benefit from their experience knowledge:

“I’ve been on the job for years and know what managing, leading and motivating means. I also know when my possibilities are exhausted, when I have reached my limit. Everything is possible if you know exactly who is responsible for what” (SN 27).

Others report benefits from their self-control and self-reflection skills. They perceive that they can control the workload by setting priorities to reduce stress for their employees. In this case, certain tasks are not being “sat out,” yet being moved down in the line of priority instead. A lack of managers’ ability to self-control can quickly lead to overwhelming the staff.

“It is essential for us to carry out prioritize. I manage many things on my own” (SN 27).

*“It’s problematic because I have a tendency to sacrifice myself. Because I can’t say no when it comes to patients. I could not prevent the illness from my secretary. Painful for me, because I know that I am part of it and could not prevent it, although I would like to have” [*…*] “There are so many requests and requirements in a hospital that need my attention and which I would like to see met. It is simply not possible, however. It is difficult to draw the line. Taking care of everyone is not easy. On the contrary, it often does not succeed at all” (CP 07).*

*“You have to be a little flexible yourself, organizational skills are important. But many people can’t do that, get sick, get job anxiety” (SN 20). “A high workload makes you somehow headless. You notice that you sometimes can’t implement your plans. Sometimes you walk a fine line: you want to be good to your employees, but you also want to take care of the tasks and demands of the nursing service management”* (SN 28).

Finally, measures with a focus on justice, appreciation, and participation of employees were mentioned. Even if there is a general lack of functioning duty schedules in hospitals, managers see possibilities for action by letting employees participate to design and to ensure a most fair work time distribution. Appreciation can be given by managers in offering trainings, feedback, new tasks or job autonomy to employees.

“I can let employees participate in creating the duty schedule so that they can express their wishes. I can try to consider their wishes by priority, so that they are somehow satisfied” (SN 22). “I have the opportunity to treat everyone equally. No matter what I think of them. I try to do that very hard. I can give someone a goodie when people step in for others. Equal treatment is very important (patient distribution, creating the duty schedule)” (SN 17).

##### External focus on managers’ behavioral control

The perception of managers’ behavioral control to implement work design measures is influenced, and often limited, by organizational factors. Restrictions for the implementation of work design measures are mainly perceived at the organizational level, partly also at the team level. Few managers perceive supportive organizational structures. The perceptions differ between occupational groups and across departments.

The managers perceive that high work intensity, the economic requirements, the lack of staff and missing job autonomy are the biggest challenges. These factors are often mentioned as difficulties to implement better working conditions by work design.

“Economic demands regulate our action’s” (CP 09). “Improvement measures cannot be implemented for financial reasons” (CP 08). “We feel that we are doing a balancing act between work intensity, the necessary overall performance in patient treatment and ensuring that employees maintain their work-ability” (CP 11).

“Changes that cost something are not popular here in the hospital. Times in healthcare have become more difficult” (SP 33).

The development of functioning team structures is experienced as challenging or impossible, especially by physicians. The cooperation in the departments is characterized by continuous staff fluctuations. Various system-related reasons are mentioned. The medical training system requires a continuous change of personnel in the department. In-house rotations of the personnel are called complicating. Illness-related absences or dismissals aggravate the situation.

“The training of medical specialists alone makes for a continuous change in the team and it’s functioning. No matter how good the functional units may be, we reach our limits in terms of personnel and economic responsibility” (CP 07).

“Too much rotation in personnel deployment makes people dissatisfied: a physician who takes the trouble today and thinks about what can be done better is somewhere else tomorrow and does not see the result. It also prevents team spirit. Our employees have no home base” (CP 013).

“I can make sure that the team function. But that has become more and more difficult in recent years. The team has changed completely since the acquisition of the new company” (SN 22).

Two chief physicians describe limitations in team development because of the strict separation between care and medicine. In general, limited possibilities to participate in the recruitment and selection of staff are described. They feel externally determined by the upper hospital management or the nursing service management and restricted in their job autonomy.

“Today, as chief physicians, we no longer have the autonomy (the position) to control a functioning unit. Today we are the foremen and are externally controlled by a superior economist. This makes it difficult to manage a functioning team from one’s point of view. Besides, as chief physicians, we lead two different questions: medical (physicians) and care (nurses). But they are not one unit. The nursing management defines how it has to work and we have limited access” (CP 05).

Regular (interdisciplinary) team communication within flat hierarchical team structures are perceived by nursing staff and senior physicians as helpful in preventing work stress. While some managers perceive organizational structures that facilitate such an exchange, other managers describe their possibilities in that respect as limited. Senior physicians mention that due to the lack of communication in the team, many problems arise that have could have been avoided. Flat hierarchies are particularly appreciated by senior physicians and nurses but still not existing, which complicate the implementation of work design measures.

“I would always wish for it, but there is no time to reflect on certain things in the team. For example, what we can do to minimize stress. We don’t realize that enough, we should do it much more often” (SP 37).

“It is really a pity that there is no real team spirit here. That would have to be strengthened. There are many more issues that have to be addressed in order to get more job satisfaction, but, unfortunately, there is no dialog for improvement. The general attitude toward improvement is very conservative” (SP 38). “Hierarchies and stuck working structures exist. This makes it difficult to change situations in a positive manner” (SP 32).

“We have a management circle in our department: the chief physician, the senior physicians and me as a senior nurse. On that board, I am able to communicate the information collected from the senior nurses. We talk about urgent things with an immediate need for action. Which might be handled in a small project. I let my colleagues participate and I thank God they join in. We also have case conferences and supervision. The communication is good, also the networking of the senior nurses works well” (SN 27).

Managers experience the greatest challenge in designing cross-departmental cooperation. Interface problems are difficult to solve and the physicians in particular often complain about the lack of cooperation with managers from other departments. Solving interface problems take time, energy, persistence, and requires suitable organizational structures to work on coordinated changes. The complexity of organizational structures in the hospital makes improvements of working conditions more difficult.

“Chief physicians do not work with the same goals in mind” (CP 01). “Many are very egocentric, and our enterprise fails because of this. What matters is the subject at work, not personal interests or vanities. The hospital extremely unorganized to the extent that one hand does not know what the other is doing” (CP 03). “Improving processes and structures takes several years. To implement measures across departments is difficult. Especially frustrating when you have tried everything. The fact that improvement often fails at the interfaces is already known (CP 02).”

In terms of this challenge, some interviewees see opportunities for chief physicians to form stronger alliances to bring across their common goals to the upper hospital management.

“Suggestions for work design would have to be bundled and sent to the employer via the chief physicians. I think that is how it would work. The departments, the chief physicians as representatives, would have to submit a consensus and ask for implementation. That would be the easiest way” (SP 33).

“If all the managers were to stand together, this would already be a great potential for cooperation with upper hospital management” (SN 23).

The cooperation with the upper hospital management is often perceived as restrictive and exhausting. Some managers point out that it takes a lot of time and effort to deal with the upper hospital management to get the problems solved. Others, who have made bad experiences in the support of the hospital management, seem resigned. They do not describe any possibilities on their own to change working conditions to the positive.

“Sometimes it’s a long-lasting struggle with the upper hospital management to get demands accepted. However, it works” (CP 15). “I have no autonomy on my own to improve the situation for employees. I have little influence on my own and can only delegate the demands from above to the bottom-up” (CP 08).

“Management by Waiting. I simply sit things out. Someone takes care of it. You’re looking forward to be free and then you keep on running in the hamster wheel. I don’t see any possibilities for me to act, I don’t have them. I can’t take the pressure out; the patients are there. Of course, I transmit the pressure” (SN 19).

On the other hand, some managers benefit from the continuity in cooperative contact with upper hospital management or other stakeholders. A good and active contact with the upper hospital management or the nursing service management does not enable the direct implementation of improvements, but there is a perception that change processes can be initiated.

“I’ve been on the job for years and know what managing, leading and motivating means. I also know when my possibilities are exhausted, when I have reached my limit. Then I can contact the upper hospital management and nursing service management that support me. You can compensate for a lot of things by reflecting on yourself. But there are also moments when you somehow need feedback, what are you doing wrong, why are you feeling so bad. We are in a department in which with me and a new deputy have many possibilities to change things. Now we are doing everything possible and have already changed a lot. Our managers give us autonomy, as long as things work. And our colleagues are invited to join us. Thank God they do” (SN 27).

## Summary and Discussion

Organizational research has shown that the support from managers is one of the key factors for the success or failure of organizational interventions (Bond and Bunce, 2001; Mattila and Elo, 2006; Nielsen et al., 2006; DeJoy et al., 2010; Bourbonnais et al., 2011; Petrou et al., 2016; Lundmark et al., 2017). However, current implementation research criticizes that success factors or obstacles for effective implementation were neglected or only considered retrospectively (Nielsen, 2013). There is a need for a theoretically sound and proactively oriented implementation research (Nielsen, 2013; Müller, 2016). With our qualitative approach and the application of the Theory of Planned Behavior (TPB) (Ajzen, 1991), we therefore explored the attitudes, organizational norms, and perceived behavioral control of managers concerning the promotion of health-related work design measures in hospitals, in order to contribute to the further theoretical and conceptional underpinning of the design and successful implementation of much needed work design interventions in hospitals.

The results on *“attitude”* show that the interviewed managers are sensitized to the topic of mental health and attach great importance to work design measures. In general, this finding indicates that the surveyed managers are willing to support health-related work design measures. However, the reasons differ depending on the occupational group: Almost all interviewees feel responsible for the mental health of their employees, but some perceive a role conflict between the fulfillment of medical and economic responsibility and the needs of the employees. Particularly, chief physicians describe the desired outcomes of work design measures from a more functional perspective (i.e., better health increases employee motivation, work ability, productivity, etc.) than the other two occupational groups (senior physicians and the senior nurses). The arguments of the latter two groups are rather based on an individual or moral health-related perspective, i.e., they consider the benefits of health promotion as a value in itself. This finding is in accordance to a study by Downey and Sharp (2007), which assumes that managers who are more under financial pressure report less moral responsibility for the employees’ health promotion than others. Even though we did not ask the question about financial pressure directly, the role of the chief physicians is in German hospitals associated with more responsibility for the budget than in the other occupational groups. Those differences between occupational groups are important, when it comes to recruiting or motivating managers for work design interventions.

*Organizational norms* are perceived very differently depending on the individual experiences of the managers and depending on their occupational group. The majority of managers do not perceive health-promoting *organizational norms*. They state that there seems to be almost no official or open dialogue on mental health promotion, neither at the organizational level nor at the departmental level. A credible and transparent positioning of the hospital management to the topic mental health promotion is mainly missing. Poor leadership styles of colleagues are criticized, departmental differences are perceived, especially by chief physicians. Few managers who have personally experienced previous support in the implementation of work design measures organizational change processes by upper hospital management describe that workplace health promotion has a high priority for the executive board. Others report the opposite. They perceive little support and sometimes appear resigned. Managers who perceive hospital management as more supportive often experience more behavioral control to implement health-related organizational measures than others. At this point, we assume a relationship between *organizational norms* and *perceived behavioral control*, which has to be examined in future studies.

In accordance with previous studies (Downey and Sharp, 2007; Wilde et al., 2011), we believe that interventions to strengthen health-promoting organizational norms can make an important contribution to ensure that managers support organizational health-promoting measures. This assumption is supported in a very recent study by Biron and Karanika-Murray (2014) which is referring to the Psychosocial Safety Climate (PSC) a specific dimension of organizational climate which describes common perceptions relating to “policies, practices and procedures for the protection of worker psychological health and safety” (Dollard and Bakker, 2010, p. 580). The study of Biron and Karanika-Murray (2014) shows that organizational factors in terms of PSC influence managers’ ownership of health-related intervention activities. Also, Biron and Karanika-Murray (2014) were able to show that the perception of support by the upper hospital management, the integration of the topic mental health in the strategic management as well as functioning communication processes are important to strengthen the managers’ commitment to health-related work design interventions.

In respect to *perceived behavioral control*, managers report that they are mainly able to provide social support, appreciation or equal rights for employees on an individual or team or departmental level. Concerning structural measures such as work design and improvement of work organization; managers perceive a rather low behavioral control. The implementation often fails due to lack of time and staff, especially due to fluctuations and absence due to illness. Cross-departmental organizational change processes are perceived as even more difficult or even impossible to implement. Success factors are considered to be an open and interdisciplinary communication culture, a common health-related goal orientation of all stakeholders, resources for the development of measures in project structures and support by hospital management. All in all, it can be assumed that work design measures are not implemented very frequently by managers. They seem to prefer individual and team related measures, focused on providing social support. Interventions to increase managers’ perception of self-efficacy and the controllability to strengthen the mental health of their employees should therefore primarily concentrate on organizational approaches to work design.

### Limitations and Future Research

Due to the voluntary participation of managers in the interview study, we cannot rule out a sampling bias. We must assume that we have primarily reached those managers who had already positive attitudes toward the topic of employees’ mental health. Moreover, participants might have shown a socially desirable response behavior. Other recruitment settings (e.g., congresses, in-house trainings) or strategies (e.g., direct letters and financial compensation) might have led to a different selection of participants.

Since we only interviewed managers of one hospital, the results cannot be generalized without further ado. The interview guideline appears to be suitable for use in other hospitals, so that its generalizability could be tested. It should be also taken into account that the results of our study only describe the perception of medical and nursing management. No conclusions can be made about how the upper hospital management really acts to improve the mental health of employees. In a follow-up study the upper hospital management also might be asked about their perception toward the topic of mental health promotion (e.g., in a focus group) and then compared with the results of the medical and nursing management.

Moreover, the study design does not allow us to make any statements about the actual behavior of managers. We decided against the assessment actual behavior for several reasons: In the context of the interviews it is difficult to make valid statements about actual behavior. Conceptually, it is difficult to separate actual behavior in the interviews from the perception of “behavioral control.” In some cases we have concluded from reported behavior on PBC. Furthermore, quantitative studies have shown that the three predictors of the TPB can predict actual behavior. In view of the reference studies and our results, we assume that managers who report more positive attitudes, perceive supportive norms and behavioral control indeed design more health-promoting working conditions for their employees. We have retrospectively examined this assumption by additionally analyzing the interviews of two managers with contrary perspectives the TPB components (Table 4). The statements in Table 4 indicate that medical and nursing managers who show higher values for the TPB components are more likely to practice health-promoting measures in work design than managers with lower values.

**TABLE 4:** Examples of managers’ statements with higher and lower TPB values.

**Mental health promotion**	**Example 1: Higher TPB values (SN 27)**	**Example 2: Lower TPB values (CP 8)**
Attitude	“This is a major issue (mental health). Because of the stress, there are always sick leaves. The bitching among each other increases, the employees walk around with grumpy faces.”	*“The topic is important for all occupational groups.” “Mental health is the responsibility of each individual. I have little influence on it.”*
Organizational Norms	*“There are not only differences in the hierarchy, but also differences in subject and occupational group specifics. In psychiatry we are much more advanced. Mental health is taken care of here.” “We are all in the same boat here. It is not about who is worse, who is better, but that we come forward together. That is already a very great cohesion here. Loyalty is also a big issue for us.”*	*“The topic of mental health is not a priority for the upper hospital management.” “There is an exchange on the level of colleagues, but more on an informal level.”*
Perceived Behavioral Control (internal)	*“I’ve been on the job for years and know what managing, leading and motivating means. I also know when my possibilities are exhausted, when I have reached my limit. Everything is possible if you know exactly who is responsible for what.” “It is essential for us to carry out prioritize. I manage many things on my own.”*	*“I have no autonomy on my own to improve the situation for employees. I have little influence on my own and can only delegate the demands from above to the bottom-up.”*
Perceived Behavioral Control (external)	*“We have a management circle in our department: the chief physician, the senior physicians and me as a senior nurse. On that board, I am able to communicate the information collected from the senior nurses. We talk about urgent things with an immediate need for action. Which might be handled in a small project. I let my colleagues participate and I thank God they join in. We also have case conferences and supervision. The communication is good, also the networking of the senior nurses works well.” “I’ve been on the job for years and know what managing, leading and motivating means. I also know when my possibilities are exhausted, when I have reached my limit. Then I can contact the upper hospital management and nursing service management that support me. You can compensate for a lot of things by reflecting on yourself. But there are also moments when you somehow need feedback, what are you doing wrong, why are you feeling so bad. We are in a department in which with me and a new deputy have many possibilities to change things. Now we are doing everything possible and have already changed a lot. Our managers give us autonomy, as long as things work. And our colleagues are invited to join us. Thank God they do.”*	*“It fails here because of too many administrative tasks and too little staff. I have no latitude to improve the situation for employees. There is no support from the upper hospital management. Improvement measures cannot be implemented due to financial restrictions.”*

Nevertheless, it would have been desirable if we could have matched the statements of the managers with the assessments of their employees regarding their actual leadership behavior.

Leadership behavior has a significant impact on the employees’ health (Kuoppala et al., 2008; Montano et al., 2017), the perception and the behavior of employees in the departments. Future quantitative research might examine the interactions between managers’ perceived organizational norms (organizational culture) and the managers’ actual behavior with regard to employee well-being and their work situation.

We are aware that all qualitative research is contextual; it takes place within a specific time and place between two or more people (Dodgson, 2019). The interviewers used a structured and pre-tested interview guide, to minimize situational or personal bias. However, complete objectivity is not given in qualitative research (Dodgson, 2019). For example, it cannot be excluded that in-depth questions differed due to personal or professional experience and interests of the interviewers.

### Conclusion and Practical Implications

Our study contributes to the research on health-related work design by showing that the theory of planned behavior (Ajzen, 1991) might be a useful theoretical framework for planning organizational interventions that are aimed to maintain and enhance the mental health of employees in hospitals. The theory can be used to capture the action guiding perceptions of managers, e.g., in relation to the implementation of health-related work design interventions. Moreover, the theory of planned behavior can be used to identify approaches for interventions that directly affect behavioral changes of managers in order to support such measures. In this way, the results of our study complement existing recommendations on how to improve the quality of the implementation of organizational interventions (Murta et al., 2007; Egan et al., 2009; Nielsen and Randall, 2012). While previous papers state that managers need to be supportive, or have even the role of “active crafters” (Nielsen, 2013) that participate in the design of organizational interventions, using the TPB allows us to understand why managers are often not willing or able to do so. Or, to put it positively: Our study allows us to better understand how to initiate the support of managers:

In respect to managers *attitudes* our findings indicate, that good treatment quality of patients and cost efficiency are often top priorities, especially for chief physicians and upper hospital management. Managers are often not aware that the health of employees is a main resource for achieving these priorities. From our point of view, managers who have not yet been sensitized to the topic of mental health promotion of employees could be reached more easily by informing them on the interrelationships between mental health, staff motivation, treatment quality, economic success and patient satisfaction (Podsakoff et al., 2007; Angerer et al., 2012; Weigl et al., 2016; Han et al., 2019). The reported role conflicts of the managers could be addressed if managers and the upper hospital management recognizes that employee health and cost efficiency are no contradictions. Moreover, when planning interventions, the different attitudes of different groups of managers should be taken into account: Chief physicians are more likely to be convinced with arguments that emphasize the link between mental health and performance. For senior physicians and nurses, moral and ethical aspects may be more important.

Our results on *organizational norms* suggest that hospitals need to establish an official and continuous dialogue on common health-related goals at the organizational or departmental level. This dialogue could be initiated by upper hospital management with the participation of upper and middle managers. Health-related company goals and offers should be developed transparently with the participation of employees. The existing management structures or instruments should be examined for possibilities to integrate health-related goals.

Our results on *perceived behavioral control* indicate that managers particularly need additional personal and external resources to implement health-related work design interventions (e.g., time, know-how). One the one hand the managers need more competencies to design work structures and processes. For example, they should be made familiar with participative approaches for healthy work design, that are based on well-founded theory and empirical evidence (e.g., Aust and Ducki, 2004; Bourbonnais et al., 2006, 2011). On the other hand, upper hospital management should provide additional external resources and accompany the development of work design measures ideally and operationally (Daniels et al., 2017). The key messages are summarized in Table 5.

**TABLE 5:** Practical approaches to foster managerial support of health-related work design interventions according the dimensions of the Theory of Planned Behavior model.

Attitude	• To reach all managers who are not yet sensitized for the issue of mental health promotion. • To reduce role conflicts, e.g., by demonstrating that employee health and performance orientation are not necessarily opposites.
Organizational Norms	• To establish a credible and transparent communication process on the importance of mental health promotion in hospitals. • To develop participative strategic and operational goals and measures to promote the mental health of employees who are integrated into existing structures.
Perceived Behavioral Control	• To develop the managers’ skills needed to implement work design measures. • To provide managers with necessary resources to implement work design measures.

Our qualitative findings might stimulate future studies that further validate our results. Moreover, our findings might further guide the development of interventions to improve health-related work design in hospitals. These measures are important to reduce the risk of impaired mental well-being among hospital staff and increase job satisfaction, which in turn have a positive effect on the quality of patient treatment.

## Author’s Note

The interview study was embedded in the cluster-randomized collaborative study “Mental Health in the workplace hospital” (SEEGEN), which aims to develop and implement behavioral and relational interventions to reduce stress in hospitals (Mulfinger et al., 2019). This project was funded by the German Federal Ministry of Education, and Research (BMBF), The University of Duisburg-Essen, Heinrich-Heine University Düsseldorf, Universities of Ulm, Tübingen, and Heidelberg are affiliated partners.

## Data Availability Statement

The data sets generated for this study will not be made publicly available. The data supporting the results of this study can be requested from MG, but restrictions apply if the use could endanger the anonymity of the participants.

## Ethics Statement

The studies involving human participants were reviewed and approved by the Ethics Committee of the Medical Faculty, University Düsseldorf. The patients/participants provided their written informed consent to participate in this study.

## Author Contributions

AM developed the study concept. MG and BW performed the data collection. MG performed the data analysis and interpretation, and drafted the manuscript. AM, PA, and BW provided the critical revision of the manuscript. All authors designed the study and approved the final version of the manuscript for submission.

## Conflict of Interest

The authors declare that the research was conducted in the absence of any commercial or financial relationships that could be construed as a potential conflict of interest.
